# Side-Clamping Versus Single Cross-Clamping in On-Pump CABG: Impact on Early Hematologic Outcomes and Blood Conservation

**DOI:** 10.3390/jcm15114124

**Published:** 2026-05-27

**Authors:** Hakan Öntaş, Asiye Aslı Gözüaçık Rüzgar

**Affiliations:** 1 Department of Cardiovascular Surgery, Balikesir University, Balıkesir 10145, Türkiye; 2 Department of Cardiovascular Surgery, Balikesir Atatürk City Hospital, Balıkesir 10100, Türkiye; drasli30@gmail.com

**Keywords:** coronary artery bypass grafting, aortic clamping, patient blood management, hemoglobin preservation, chest tube drainage

## Abstract

Aortic manipulation and prolonged ischemia during cardiopulmonary bypass remain major drivers of perioperative complications. Optimizing clamping strategies is crucial to minimize iatrogenic blood loss and improve recovery profiles within a Patient Blood Management (PBM) framework. **Objectives**: This study aimed to evaluate the impact of two different aortic clamping strategies—single cross-clamping versus side-clamping—on early hematologic outcomes within a Patient Blood Management (PBM) framework in patients undergoing on-pump coronary artery bypass grafting (CABG). **Methods**: In this retrospective cohort study, 265 patients who underwent elective isolated 3-vessel on-pump CABG between January 2019 and April 2025 were analyzed. Patients were divided into two groups: the Side-Clamp group (*n* = 132), where proximal anastomoses were performed under partial occlusion after releasing the main cross-clamp, and the Single Cross-Clamp group (*n* = 133), where all anastomoses were completed during a single arrest period. The primary outcomes were postoperative hemoglobin (Hb) drop and 24-h chest tube drainage. **Results**: The side-clamp technique was associated with significantly shorter cardioplegic arrest times (34.5 ± 3.7 vs. 52.0 ± 3.9 min; *p* < 0.001). Key findings revealed that the postoperative hemoglobin drop was significantly lower in the side-clamp group (1.9 ± 0.4 g/dL vs. 2.1 ± 0.4 g/dL; *p* = 0.005). Notably, the side-clamp group also exhibited significantly lower 24-h chest tube drainage (123.4 ± 28.3 mL vs. 131.0 ± 27.4 mL; *p* = 0.027). Multivariate linear regression analysis, adjusting for age, sex, and CPB time, confirmed the side-clamp technique as an independent predictor of superior hemoglobin preservation (β = −0.115, *p* = 0.009). No significant differences were observed regarding the incidence of postoperative AF (13.6% vs. 15.8%; *p* = 0.748) or 30-day mortality. **Conclusions**: The side-clamp strategy is associated with modest but statistically significant reductions in postoperative hemoglobin decline and 24-h chest tube drainage. While these differences did not translate into a significant reduction in RBC transfusion or early mortality, the side-clamp technique represents a useful technical component within a broader Patient Blood Management strategy in routine on-pump CABG practice.

## 1. Introduction

Coronary artery disease (CAD) remains the leading cause of global morbidity and mortality [1]. Coronary artery bypass grafting (CABG) continues to be the most established surgical strategy for myocardial revascularization in advanced CAD, effectively relieving ischemic symptoms and improving long-term survival. Despite its benefits, CABG involves significant physiological challenges primarily related to cardiopulmonary bypass (CPB) and aortic manipulation, with intraoperative clamp and CPB durations being well-established determinants of postoperative morbidity and mortality [2]. Prolonged CPB exposure contributes to ischemia–reperfusion injury and systemic inflammatory responses, where elevated postoperative lactate levels serve as early biochemical markers of poor clinical outcomes [3,4]. Recent evidence suggests that the discrepancy between total CPB time and aortic cross-clamp time is a potent predictor of adverse events, highlighting that the balance between extracorporeal circulation and ischemic arrest is highly clinically relevant [5]. Furthermore, prolonged cross-clamping, especially exceeding 60 min, intensifies oxidative stress and remains an independent risk factor for impaired ventricular performance and overall morbidity [6,7].

In parallel with these ischemic concerns, contemporary cardiac surgery has increasingly shifted its focus toward Patient Blood Management (PBM)—a multimodal, evidence-based approach designed to optimize patient outcomes by preserving endogenous hemoglobin and minimizing iatrogenic blood loss. In the context of on-pump CABG, perioperative anemia, surgical blood loss, and hemodilution are central challenges that influence early recovery. While the impact of clamping durations is well-documented, the influence of the specific clamping strategy on the hematologic profile remains an underexplored facet of PBM.

The choice of aortic clamping method for performing proximal anastomoses represents a critical decision point in surgical strategy. In the single cross-clamp approach, both distal and proximal anastomoses are completed under a single period of cardioplegic arrest. Conversely, in the side-clamp (partial occlusion) approach, proximal anastomoses are constructed on a beating or vented heart after the release of the main cross-clamp. Traditionally, the debate between these techniques has centered on myocardial protection and neurological safety. However, in the era of PBM, the potential of these strategies to influence surgical blood loss, perioperative hemoglobin stability, and postoperative arrhythmias warrants rigorous investigation.

Purpose and Rationale This study addresses a critical analytical gap by evaluating the association between aortic clamping strategy and early hematologic outcomes in a cohort of 265 patients undergoing elective on-pump CABG. The primary objective is to determine whether the side-clamp or single cross-clamp strategy offers superior results regarding postoperative hemoglobin preservation, 24-h chest tube drainage, and the incidence of atrial fibrillation (AF). By focusing on these parameters within a standardized surgical setting, we aim to provide a clinical framework for optimizing blood conservation and enhancing surgical safety in routine CABG practice.

## 2. Materials and Methods

### 2.1. Study Design and Ethics

This retrospective cohort study was conducted at Balikesir Atatürk City Hospital, analyzing patients who underwent isolated on-pump coronary artery bypass grafting (CABG) between January 2019 and April 2025. The study protocol was approved by the Institutional Review Board of Balikesir Atatürk City Hospital (Approval Code: 2025/10/116; Date: 23 October 2025) and adhered to the principles of the Declaration of Helsinki.

### 2.2. Study Population and PBM Framework

The study population consisted of 265 consecutive patients who underwent isolated 3-vessel CABG using the left internal mammary artery (LIMA) and saphenous vein grafts. To evaluate the impact of surgical technique on Patient Blood Management (PBM), patients were categorized into two groups based on the proximal anastomosis strategy:

Side-Clamp Group (*n* = 132): Proximal anastomoses were performed on a beating heart using a partial occlusion clamp after releasing the main cross-clamp.

Single Cross-Clamp Group (*n* = 133): All distal and proximal anastomoses were completed during a single period of cardioplegic arrest.

All patients were managed according to a standardized institutional PBM protocol to ensure consistency across study groups. Preoperative antiplatelet therapy was managed by continuing aspirin while ceasing clopidogrel at least five days prior to elective surgery. Intraoperative cardiopulmonary bypass (CPB) management included routine cell salvage and maintaining a minimum hematocrit target of 21–24% during extracorporeal circulation. Heparin reversal was achieved with protamine sulfate at a 1:1 ratio. Postoperative red blood cell (RBC) transfusion was governed by a restrictive strategy, with a threshold of hemoglobin < 8 g/dL, or earlier in cases of hemodynamic instability, active bleeding, or clinical evidence of inadequate oxygen delivery. The same PBM protocol was rigorously applied to both study groups throughout the study period.

Inclusion Criteria:

Patients aged > 18 years undergoing elective isolated on-pump CABG.
Presence of angiographically proven 3-vessel coronary artery disease.

Exclusion Criteria:

Emergency or urgent surgical procedures.
Combined valvular, aortic, or non-isolated operations.
Preoperative anemia defined as a baseline hemoglobin level < 10 g/dL.
Severe renal dysfunction (Chronic Kidney Disease Stage 4–5).
History of previous cardiac surgery (redo-CABG cases).

### 2.3. Surgical Techniques and Myocardial Protection

**Figure 1:**
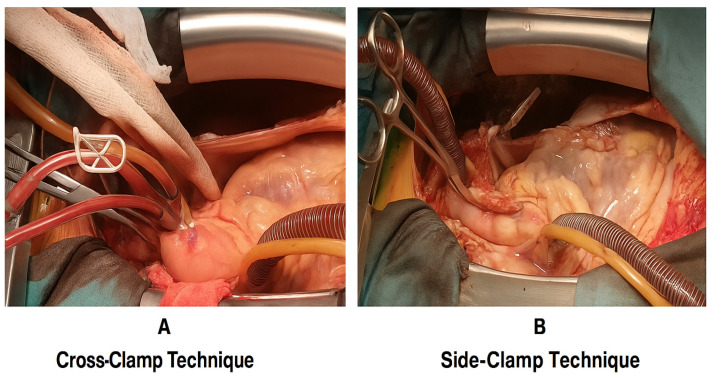
Aortic clamping techniques during cardiopulmonary bypass (On-Pump).

All procedures were performed via median sternotomy under cardiopulmonary bypass (CPB) with mild hypothermia (32 °C). Myocardial protection was standardized using del Nido cardioplegia. The comparison of the two aortic clamping techniques used for proximal anastomoses during on-pump CABG is illustrated in Figure 1.

**(A)**  **Single Cross-Clamping:** The aorta was cross-clamped once, and both distal and proximal anastomoses were completed during a single period of cardioplegic arrest. The clamp was removed only after all sutures were finalized.
**(B)**  **Side-Clamping:** Distal anastomoses were performed under cross-clamping. Following the delivery of warm cardioplegia, the cross-clamp was removed, allowing the heart to resume beating. Proximal anastomoses were then constructed using a side-clamp (partial occlusion).

### 2.4. Data Collection and Hematologic Outcomes

The primary focus of this study was early hematologic outcomes within the PBM framework. Perioperative data were extracted from the institutional electronic database.

Hematologic Parameters: These included preoperative hemoglobin (Hb), postoperative 24-h Hb, and the total hemoglobin drop (ΔHb).

Clinical Outcomes: Twenty-four-hour chest tube drainage (mL), requirement for red blood cell (RBC) transfusion, and the incidence of postoperative atrial fibrillation (AF) and stroke/TIA were recorded.

The primary hematologic endpoint, hemoglobin drop (ΔHb), was defined as the absolute difference between the preoperative hemoglobin level and the hemoglobin value measured 24 h after surgery. This specific timeframe was utilized to standardize the evaluation of early postoperative hematologic recovery and to minimize the potential confounding effects of late-phase fluid shifts or varying transfusion timings.

### 2.5. Statistical Analysis

Statistical analysis was performed using SPSS version 26.0. The normality of continuous variables was assessed using the Shapiro–Wilk test. Continuous variables were expressed as mean ± standard deviation, and categorical variables as frequencies and percentages. A single multivariable linear regression model was constructed to identify independent predictors of postoperative hemoglobin drop (dependent variable). Variables entered into the model included clamping strategy (coded as 0 for Single Cross-Clamp and 1 for Side-Clamp), age, sex, CPB time, preoperative hemoglobin level, and previous myocardial infarction. Multi-collinearity was assessed using the Variance Inflation Factor (VIF), with a VIF < 5 considered acceptable. A *p*-value of <0.05 was considered statistically significant.

### 2.6. Use of Artificial Intelligence

Generative AI tools ChatGPT 5.1 and Gemini Pro were used solely for language editing and grammatical refinement of the manuscript.

## 3. Results

### 3.1. Baseline Characteristics

The study analyzed 265 patients, with 132 in the Side-Clamp group and 133 in the Single Cross-Clamp group. Preoperative demographic and clinical characteristics are summarized in Table 1. The cohorts were generally well-matched; however, patients in the Cross-Clamp group were slightly older (65.3 ± 6.5 vs. 63.6 ± 6.5 years; *p* = 0.036). A significantly higher proportion of patients in the side-clamp group had a history of previous myocardial infarction (38.6% vs. 18.8%; *p* = 0.001). No significant differences were observed regarding BMI, diabetes mellitus, hypertension, or preoperative hemoglobin levels (13.3 ± 0.9 vs. 13.4 ± 0.8 g/dL; *p* = 0.293).

### 3.2. Clinical and Postoperative Results

Operative data and early clinical outcomes are presented in Table 2. As per the technical definition, the Side-Clamp group had significantly shorter cardioplegic arrest (clamp) times (34.5 ± 3.7 vs. 52.0 ± 3.9 min; *p* < 0.001). Total operative time and CPB time were statistically comparable between the groups.

Regarding hematologic outcomes, the hemoglobin drop (ΔHb) was significantly lower in the Side-Clamp group (1.9 ± 0.4 g/dL) compared to the Cross-Clamp group (2.1 ± 0.4 g/dL) (*p* = 0.005). Furthermore, the Side-Clamp group exhibited significantly lower 24-h chest tube drainage (123.4 ± 28.3 vs. 131.0 ± 27.4 mL; *p* = 0.027). The incidence of postoperative AF was numerically lower in the Side-Clamp group (13.6% vs. 15.8%), though this difference did not reach statistical significance (*p* = 0.748). Mortality and RBC transfusion rates were similar between groups.

### 3.3. Multivariate Analysis

Since baseline differences were observed in age and previous MI, a multivariable linear regression model was constructed to identify independent predictors of postoperative hemoglobin drop. After adjusting for clamping strategy, age, sex, CPB time, preoperative hemoglobin levels, and previous myocardial infarction, the side-clamp technique remained a significant independent predictor of superior hemoglobin preservation (β = −0.115, 95% CI: −0.20 to −0.03, *p* = 0.009; Table 3).

Additionally, age (95% CI: 0.01 to 0.03; *p* = 0.001) and CPB time (95% CI: 0.005 to 0.015; *p* = 0.001) were identified as independent factors significantly influencing the magnitude of hemoglobin decline. In a separate logistic regression model, the clamping strategy was not found to be an independent predictor of postoperative AF (*p* = 0.769).

## 4. Discussion

The findings of this study provide significant evidence for the role of aortic clamping strategies within the framework of Patient Blood Management (PBM) in cardiac surgery. While previous research has often focused on the side-clamp technique’s advantages in off-pump surgery, our data demonstrate that side-clamping offers superior perioperative hemoglobin preservation and a significant reduction in chest tube drainage even in the on-pump setting. With a robust cohort of 265 patients, our study highlights that technical modifications in proximal anastomosis can independently mitigate the “lethal triad” of anemia, bleeding, and transfusion [8].

A central finding of our analysis is the significantly lower hemoglobin drop (1.9 ± 0.4 vs. 2.1 ± 0.4 g/dL; *p* = 0.005) and reduced 24-h chest tube drainage (123.4 ± 28.3 vs. 131.0 ± 27.4 mL; *p* = 0.027) in the side-clamp group. Unlike our previous preliminary observations, this larger dataset confirms that reducing the total duration of complete aortic occlusion not only mitigates global myocardial ischemia but also contributes to better surgical blood management. Durmaz and Gündöner reported a correlation between aortic cross-clamp time and inflammatory cascades that trigger postoperative bleeding [9]. Our results support this mechanism: by releasing the main cross-clamp earlier and performing proximal anastomoses under partial occlusion, we likely attenuate the systemic inflammatory response and the associated coagulopathy, leading to significantly lower drainage volumes.

The multivariable regression analysis further strengthens our conclusions and confirms the robustness of the surgical strategy’s impact. After adjusting for critical confounding factors—including age, sex, CPB time, and particularly previous myocardial infarction—the side-clamp technique remained a significant independent predictor of a lower hemoglobin drop (β = −0.115$, 95% CI: −0.20 to −0.03, *p* = 0.009). This reinforces that the hematologic benefits of reduced ischemic arrest are independent of the patients’ baseline clinical history. Maintaining higher endogenous hemoglobin levels through this technical modification aligns directly with the ‘pillar one’ of PBM—optimizing red cell mass and minimizing iatrogenic blood loss. While the absolute difference in hemoglobin decline was modest, even such preservation is a critical clinical objective, as perioperative anemia is a recognized factor that can influence recovery trajectories in cardiac surgery.

Regarding postoperative atrial fibrillation (POAF), our study found no statistically significant difference between the two techniques (*p* = 0.748), suggesting that POAF is more closely linked to total CPB duration and systemic inflammatory markers than to the specific clamping method used for proximal anastomoses. This finding is consistent with Dayi and Çalık, who identified CPB-induced oxidative stress as the primary driver of arrhythmias [10]. While Khajeh and Zarrabi suggested that delicate aortic manipulation could reduce AF incidence [11], our data indicate that any potential benefit of side-clamping for AF may be neutralized by the mechanical stress of applying a secondary clamp on the aorta.

Aortic manipulation risks, particularly the risk of stroke, must always be considered when using side-clamps in calcified aortas [12,13]. In our cohort, we observed no significant difference in neurological events, with a stroke rate of 0.8% in both groups (*p* = 1.000). While this suggests that the side-clamp technique is safe in elective cases with appropriate aortic assessment, the neurological safety of partial occlusion clamping should be further validated in studies specifically designed to assess aortic atheroma burden.

In recent years, novel biomarkers, such as the Systemic Immune-Inflammation Index (SII), have demonstrated that prolonged aortic cross-clamp time is directly correlated with an augmented systemic inflammatory response [9]. In parallel, innovative modifications in proximal anastomosis techniques, such as sequential partial clamping, are being investigated to reduce myocardial injury and lower the incidence of postoperative arrhythmias [14]. However, the debate regarding the relative safety between side-clamping and cross-clamping continues, particularly concerning the balance between neurological risks and hemodynamic stability [15].

Taken together, these findings suggest that aortic clamping contributes to postoperative morbidity not only through mechanical ischemia but also via inflammation-mediated mechanisms. Although advanced techniques like sequential partial clamping aim to attenuate this response [16], the relative contributions of different clamping strategies to local effects (e.g., bleeding), CPB duration, and inflammation-mediated systemic effects remain incompletely defined [17].

This study has several limitations that warrant consideration. First, its retrospective and non-randomized design introduces the possibility of selection bias, as the choice of clamping strategy may have been influenced by surgeon preference or intraoperative assessment of the ascending aorta. Second, although the groups were generally comparable, baseline differences were observed in age and previous myocardial infarction. Third, the absolute differences in hemoglobin decline (approximately 0.2 g/dL) and 24-h chest tube drainage (approximately 7.6 mL) were clinically modest and did not translate into a statistically significant reduction in major postoperative complications or resource utilization, such as RBC transfusion, postoperative AF, stroke/TIA, or 30-day mortality. Fourth, detailed coagulation parameters, inflammatory biomarkers, and ROTEM/TEG data were not available, which limits a deeper mechanistic interpretation of the observed hematologic benefits. Due to sample size constraints within our single-center retrospective cohort, a propensity-score matching analysis was not feasible; however, a robust multivariable linear regression was utilized to systematically adjust for baseline clinical variations, including age and previous myocardial infarction history. Furthermore, detailed perioperative inflammatory biomarkers (such as C-reactive protein or fibrinogen) and advanced point-of-care coagulation profiles (ROTEM/TEG) were not systematically recorded in our retrospective database, which limits a deeper mechanistic interpretation of the observed hematologic benefits. Finally, the single-center design may limit the generalizability of the findings to different surgical settings or Patient Blood Management protocols.

## 5. Conclusions

In elective isolated on-pump CABG, the side-clamp strategy was associated with shorter cardioplegic arrest time and modest but statistically significant reductions in postoperative hemoglobin decline and 24-h chest tube drainage. However, these early hematologic differences were minor in absolute terms and did not result in significant reductions in major clinical endpoints, including RBC transfusion, postoperative atrial fibrillation, stroke/TIA, or 30-day mortality. Therefore, the side-clamp technique should be viewed as a useful technical component within a comprehensive Patient Blood Management framework rather than a standalone clinical solution, and its clinical relevance requires further confirmation in larger prospective trials.

## Figures and Tables

**Table 1:** Preoperative demographic characteristics of patients.

Variable	Side-Clamp (*n* = 132)	Cross-Clamp (*n* = 133)	*p*-Value
Age (years)	63.6 ± 6.5	65.3 ± 6.5	0.036 *
Sex (Male)	85 (64.4%)	99 (74.4%)	0.101
BMI (kg/m^2^)	26.3 ± 3.1	26.4 ± 3.1	0.824
Diabetes Mellitus	52 (39.4%)	47 (35.3%)	0.579
Hypertension	71 (53.8%)	78 (58.6%)	0.501
Previous MI	51 (38.6%)	25 (18.8%)	0.001 *
LVEF (%)	52.6 ± 5.0	51.7 ± 6.1	0.206
Preop Hemoglobin (g/dL)	13.3 ± 0.9	13.4 ± 0.8	0.293

* *p* < 0.05 is considered statistically significant.

**Table 2:** Operative and postoperative outcomes †.

Variable	Side-Clamp (*n* = 132)	Cross-Clamp (*n* = 133)	*p*-Value
CPB Time (min)	78.5 ± 9.2	79.3 ± 9.4	0.474
Clamp Time (min)	34.5 ± 3.7	52.0 ± 3.9	<0.001 *
Total Op. Time (min)	167.0 ± 12.4	168.5 ± 10.6	0.283
Hemoglobin Drop (g/dL)	1.9 ± 0.4	2.1 ± 0.4	0.005 *
24-h Drainage (mL)	123.4 ± 28.3	131.0 ± 27.4	0.027 *
Postop AF, *n* (%)	18 (13.6%)	21 (15.8%)	0.748
RBC Transfusion, *n* (%)	15 (11.4%)	20 (15.0%)	0.483
30-day Mortality, *n* (%)	4 (3.0%)	5 (3.8%)	1.000
Postoperative Stroke/TIA, *n* (%)	1 (0.8%)	1 (0.8%)	1.000

† Values are expressed as mean ± SD or *n* (%). AF: Atrial Fibrillation. * Statistically significant (*p* < 0.05).

**Table 3:** Multivariable linear regression analysis for postoperative hemoglobin drop.

Variable	Beta Coefficient (β)	95% CI	*p*-Value	VIF
Clamping Strategy (Side vs. Single)	−0.115	−0.20 to −0.03	0.009	1.12
Age	0.020	0.01 to 0.03	0.001	1.08
CPB Time	0.010	0.005 to 0.015	0.001	1.25
Preoperative Hb	−0.050	−0.12 to 0.02	0.150	1.15
Previous MI	0.030	−0.04 to 0.10	0.450	1.10

## Data Availability

The data presented in this study are available on request from the corresponding author. The data are not publicly available due to privacy and ethical restrictions.

## Abbreviations

The following abbreviations are used in this manuscript:

AF	Atrial Fibrillation
CABG	Coronary artery bypass grafting
CAD	Coronary artery disease
CPB	Cardiopulmonary bypass
POAF	Postoperative atrial fibrillation
BMI	Body Mass Index
LVEF	Left ventricular ejection fraction
MI	Myocardial infarction
DM	Diabetes Mellitus
HT	Hypertension
SIII	Systemic Immuno-Inflammation Index
